# Quorum Quenching *Bacillus sonorensis* Isolated from Soya Sauce Fermentation Brine

**DOI:** 10.3390/s120404065

**Published:** 2012-03-27

**Authors:** Wai-Fong Yin, Hun-Jiat Tung, Choon-Kook Sam, Chong-Lek Koh, Kok-Gan Chan

**Affiliations:** 1 Division of Genetics and Molecular Biology, Institute of Biological Sciences, Faculty of Science, University of Malaya, 50603 Kuala Lumpur, Malaysia; E-Mails: yinwaifong@yahoo.com (W.-F.Y.); jiatth@yahoo.com (H.-J.T.); 2 Natural Sciences and Science Education AG, National Institute of Education, Nanyang Technological University, 1 Nanyang Walk, Singapore 637616, Singapore; E-Mails: choonkook.sam@nie.edu.sg (C.-K.S.); chonglek.koh@nie.edu.sg (C.-L.K.)

**Keywords:** *Bacillus sonorensis*, *N*-acylhomoserine lactone, quorum quenching, quorum sensing, soya sauce fermentation brine

## Abstract

An *N*-acylhomoserine lactone (AHL)-degrading bacterial strain, L62, was isolated from a sample of fermentation brine of Chinese soya sauce by using rich medium agar supplemented with soya sauce (10% v/v). L62, a rod-shaped Gram positive bacterium with amylolytic activity, was phylogentically related to *Bacillus sonorensis* by 16S ribosomal DNA and *rpoB* sequence analyses. *B. sonorensis* L62 efficiently degraded *N*-3-oxohexanoyl homoserine lactone and *N*-octanoylhomoserine lactone. However, the *aiiA* homologue, encoding an autoinducer inactivation enzyme catalyzing the degradation of AHLs, was not detected in L62, suggesting the presence of a different AHL-degrading gene in L62. To the best of our knowledge, this is the first report of AHL-degrading *B. sonorensis* from soya sauce liquid state fermentation.

## 1. Introduction

Gram negative bacterial communication achieved via production and sensing of freely diffusible *N*-acylhomoserine lactone (AHL) molecules is known as quorum sensing (QS) [1]. The *N*-acyl side chains of different AHLs may vary in length, saturation, and substitution [2]. AHLs are synthesised by LuxI homologue synthase. Upon reaching threshold level, AHLs will bind to their cognate receptor (LuxR homologue) and affect several QS-dependent phenotypes. QS regulates a broad range of biological activities, including luminescence, antibiotic production, plasmid transfer, and virulence [3–5]. It has been shown that QS also plays significant role in food related bacteria [6].

Quorum quenching (QQ) refers to the process of interrupting QS and has been regarded as a promising novel approach to attenuate bacterial pathogens [3]. Enzymatic inactivation of AHL can be through either the opening of the lactone ring moiety by AHL lactonase or detachment of *N*-acyl side chain from the lactone ring via acylase [7–9].

Chinese soya sauce is widely used as a flavour enhancer in Asia, including China, Japan, and Southeast Asia. It is a liquid condiment employed in most oriental cuisine owing to its unique characteristic taste and it can be served plain as a sauce or form part of the cooking ingredients. Chinese soya sauce is produced by a two-stage fermentation process, namely solid state and liquid fermentations. In solid state fermentation, cooked soya beans are mixed with an arbitrary amount of flour to allow *Aspergillus oryzae* or *Aspergillus sojae* to grow on the materials at room temperature. However, brine fermentation involves the concurrent fermentation of soya mash in sea salt brine (17–20% w/v NaCl) with added bacteria and yeasts [10]. After a specific period of aging, the final product is made by pressing the soya mash and then filtered, before sugar is added to taste. Soya sauce is then sold as extra virgin for the first extraction. A subsequent extraction is achieved via extracting the second liquid fermentation after addition of more sea salt brine to the extracted soya mash and fermentation in the similar manner as described. The second extract will be sold as lower grade soya sauce.

To date, no QQ bacteria have been reported from fermented brines used to make soya sauce. To address this issue, we isolated culturable bacterium from a commercial well-aged (three months) soya sauce mash during brine fermentation. Subsequently, we characterised a novel amylolytic, QQ bacterium *Bacillus sonorensis* L62 that efficiently degraded AHLs *in vitro*.

## 2. Experimental Section

In addition to the bacterial strains isolated from soya sauce fermentation brine, bacteria used in this study included *Escherichia coli* DH5α, *Bacillus cereus* [11], and the biosensor *Chromobacterium violaceum* CV026 which detects short chain AHLs [12].

Lysogeny broth (LB) rich medium (per 100 mL: 1.0 g tryptone, 0.5 g yeast extract, 0.5 g NaCl) was used. To prepare modified LB medium (LBm) supplemented with different soya sauce concentrations, the following compositions were used (per 100 mL): 1.0 g tryptone, 0.5 g yeast extract, 0.5 g NaCl, supplemented with appropriate volumes of soya sauce at 5, 10, and 15% (v/v). The media were autoclaved. Bacteriological agar (1.5% w/v) was used to solidify LB and LBm. For selection of transformants, LB agar was supplemented with ampicillin (100 μg/mL).

Starch agar was prepared according to Stark *et al.* [13], with the following composition (per 100 mL): 0.5 g soluble starch (BDH Chemical Ltd., Poole, UK), 0.2 g yeast extract, 0.5 g tryptone, 0.5 g NaCl, and 1.5 g bacteriological agar. Bacterial colonies were streaked onto the starch agar and incubated for 24 to 72 h at 37 °C. Amylolytic activity was detected by the formation of halos around bacterial colonies after flooding the agar with iodine solution.

A well-aged (three months) sample of soya sauce fermentation brine (100 mL) was collected at 0.5 cm below the surface of a liquid state fermentation in a fermentation tank of a local factory. Aliquots of the sample (100 μL) were spread on LB and LBm plates, which were subsequently incubated at 37 °C for 24 to 48 h. Pure colonies were obtained by repeated dilution streaking on LB agar. For routine maintenance, bacterial isolates were kept on LB agar slants and in glycerol (80% v/v) at −80 °C.

Microbiological and molecular techniques, as described previously [14], were performed to identify one of the bacterial isolates, L62. Gram staining was performed and bacterial cell morphology was observed using light microscope (Olympus, Japan) at 1,000× magnification. To obtain the 16S ribosomal DNA (rDNA) of L62, an internal fragment of ≈1.5 kb was amplified by the polymerase chain reaction (PCR) with bacterial genomic DNA as template. The 16S rDNA PCR primers 27F (5′-AGAGTTTGATC(M)TGGC-TCAG-3′) and 1525R (5′-AAGGAGGTG(W)TCCA(R)-CC-3′) were used as forward and reverse primers, respectively. The *rpoB* gene, encoding the β-subunit of RNA polymerase, of L62 was amplified with reported primers rpoBF (5′-AGGTCAACTAGTTCAGTA TGGACG-3′) and rpoBR (5′-ACCGTAACCGGCAACTTAC-3′) as described by Palmisano *et al.* [15]. Purification, ligation, transformation, and sequencing of PCR products were carried out essentially as previously described [16]. Phylogenetic analysis was done by the Neighbor-Joining method using MEGA version 4.0 [17] as reported elsewhere [16].

Bacterial cells (5 mL) were grown in LB broth at 37 °C (220 rpm) to stationary phase. Cells were collected by centrifugation, washed twice, and suspended in phosphate buffered saline (PBS, 150 mM, pH 6.5). The resulting concentrated cell suspension was used as the source of resting cells for *in vitro* AHL inactivation assays, as previously described [14]. The AHLs tested were *N*-3-oxohexanoyl homoserine lactone (3-oxo-C6-HSL), *N*-heptanoylhomoserine lactone (C7-HSL) and *N*-octanoylhomoserine lactone (C8-HSL) (Sigma-Aldrich, St. Louis, MO, USA). For the whole-cell assay, 5 μL of AHL in absolute ethanol was dispensed into a sterile tube and the solvent evaporated to dryness. The dried AHL was then rehydrated with 100 μL of a bacterial cell suspension to a final AHL concentration of 0.5 μg/μL. The resting cell suspension was incubated at 37 °C for up to 24 h and at regular intervals (0, 6, 18, and 24 h), aliquots (15 μL) were withdrawn and heat inactivated (95 °C, 3 min). Experiments involving *E. coli* DH5α and PBS served as negative controls and *B. cereus* was included as a positive control for AHL-degradation. Residual AHLs were detected by the formation of purple pigmentation on *C. violaceum* CV026 lawn [14].

Rapid Resolution Liquid Chromatography (RRLC) analysis was carried out to further confirm the QQ activity of L62. To analyze the degradation of AHLs over a period of time, we used an RRLC instrument (Agilent Technologies 1200 series) equipped with an Agilent ZORBAX Eclipse^®^ XDB-C18 column (4.6 × 50 mm, 1.8 μm particle size) as reported [14]. The elution procedure consisted of an isocratic profile of acetonitrile-water (35:65, v/v) for 3 min at a constant flow rate of 0.7 mL/min and monitored at 210 nm. Both the retention time and spectral properties were compared to those of synthetic AHL standards. AHLs incubated with washed *E. coli* DH5α cells and PBS were used as negative controls.

The 16S rDNA and *rpoB* gene sequences of L62 (GenBank accession numbers of HM191249 and HQ108343, respectively) have been deposited at GenBank.

## 3. Results and Discussion

### 3.1. Isolation of L62 from Soya Sauce Fermentation Brine

**Figure 1.**
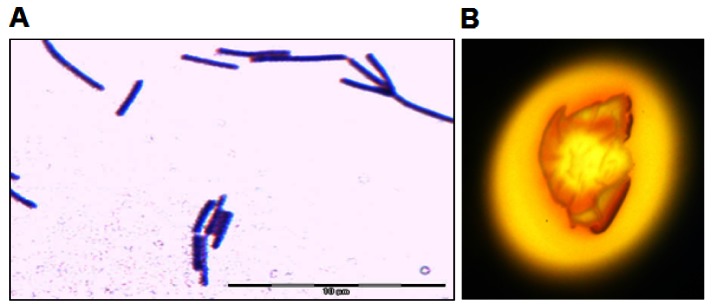
Morphology and amylolytic activity of L62. (**A**) Cell morphology of L62 (at 1,000× magnification using light microscope). Bar represents 10 μm. (**B**) Amylolytic activity of strain L62, as indicated by the halo formation surrounding the bacterial colony.

Isolate L62 was isolated from a sample of soya sauce fermentation brine plated on LBm (10% v/v soya sauce). Bacterial isolates were then streaked on LBm agar repeatedly to obtain pure colonies, which appeared after incubation for 24 to 48 h at 37 °C. The bacterial colonies were morphologically homogeneous, suggesting that limited types of bacteria were enriched in LBm. When grown on LB agar, its colonies appeared brown yellowish and irregular with undulate margin. Its rod-shaped cells were Gram stain positive (Figure 1(A)). L62 showed amylolytic activity after incubation for 72 h (Figure 1(B)).

### 3.2. Molecular Characterisation of L62

**Figure 2.**
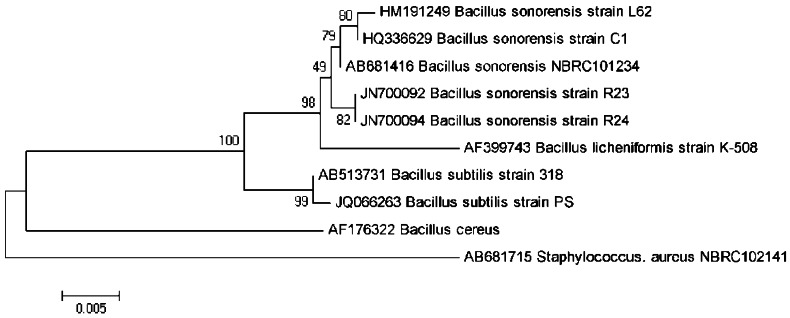
Phylogenetic analysis of the 16S rDNA gene of L62. Bootstrap values (expressed as percentages of 1,000 replications) are shown at branch points. Bar represents evolutionary distances as 0.005 changes per nucleotide position. *Staphylococcus aureus* NBRC102141 served as outgroup. GenBank accession numbers are shown on the left of species names.

The 16S rDNA gene of isolate L62 was PCR amplified, sequenced, edited, aligned, and matched against similar sequences in the GenBank. Its 1,542 nucleotides showed 99.8% similarity with the 16S rDNA of *B. sonorensis* strain C1 (GenBank accession number HQ336629). A phylogenetic tree was constructed based on the 16S rDNA gene sequences of L62, other related bacterial species, and an outgroup (Figure 2). Strain L62 is phylogenetically related to *B. sonorensis* as compared to other bacilli. Furthermore, the amplification and analysis of the *rpoB* gene confirmed our identification of isolate L62 to be *B. sonorensis* (data not shown). Hence, L62 was named *B. sonorensis* L62.

### 3.3. AHL-Degradation by Isolate L62

**Figure 3.**
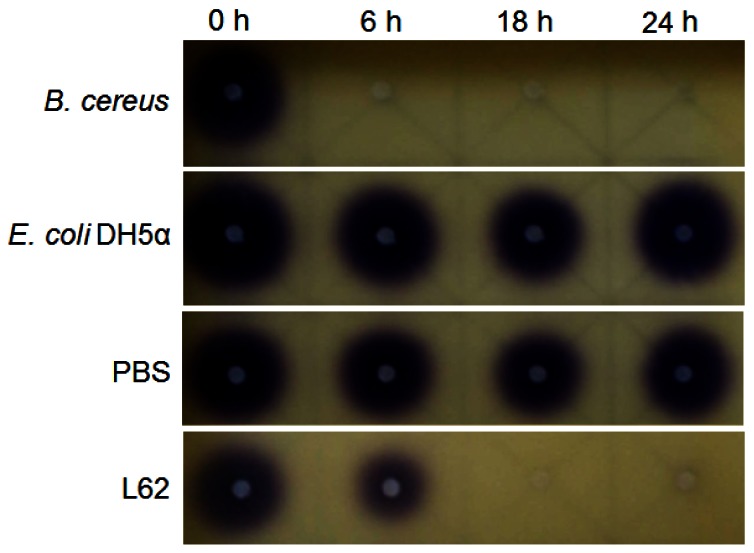
Degradation of 3-oxo-C6-HSL by *B. sonorensis*. Disappearance of 3-oxo-C6-HSL was revealed by decreased or loss of purple pigmentation on the biosensor *C. violaceum* CV026 lawn. From left to right, degradation of 3-oxo-C6-HSL investigated at 0, 6, 18, and 24 h. Rows from top to bottom: *B. cereus* (positive control), *E. coli* DH5α (negative control), PBS (extraction buffer, negative control), and L62 (*B. sonorensis*).

**Figure 4.**
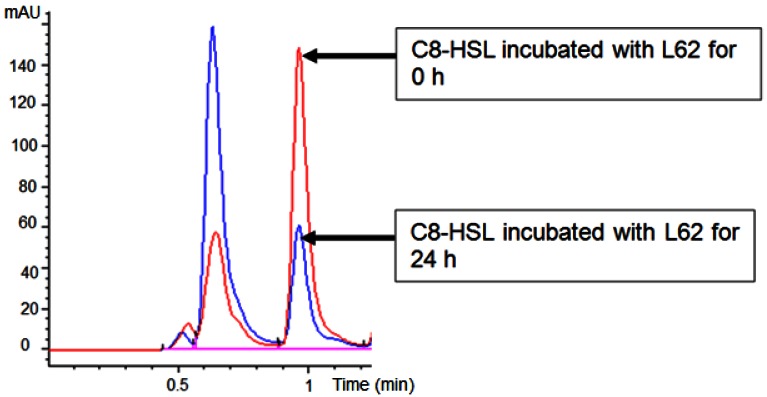
RRLC analysis of C8-HSL after incubation with *B. sonorensis* L62 resting cells for 0 (red) and 24 h (blue). Note the degradation of C8-HSL after incubation for 24 h corresponded to the drop of peak (with retention time (RT) at 0.95 min in the RRLC chromatogram). Preceding peak (RT = 0.65 min) was solvent front. mAU: Absorbance unit at 210 nm.

Isolate L62 degraded 3-oxo-C6-HSL efficiently within 6 h and no detectable AHL molecules were found after further incubation to 18 h (Figure 3). No significant C7-HSL degradation by isolate L62 was observed (data not shown). To further investigate AHL-degradation specificity, we incubated PBS-washed L62 cells with C8-HSL and used RRLC to analyze the resulting growth medium. Based on a published criterion [16], Figure 4 shows that L62 degraded about 60% of C8-HSL within 24 h, suggesting that L62 could inactivate AHL molecules with different substitutions at C3 position and with C6 and C8 *N*-acyl side chains. No apparent C8-HSL degradation was observed when the same experiment was repeated with the incubation buffer (PBS) and *E. coli* DH5α cells serving as negative controls (data not shown).

Recently, a number of food-related bacteria have been reported to show QS activity [6]. However, no report has indicated the presence of AHL-degrading bacteria in the unique fermentation of soya sauce. Here, we describe a QQ positive bacterial isolate, *B. sonorensis* L62, from soya sauce fermentation brine. L62 grew well on LBm (10% v/v soya sauce) and LB agar.

*B. sonorensis*, first isolated from desert soil, has been reported by Palmisano *et al.* [15] to withstand highly saline soils and dehydration. Later, *B. sonorensis* has also been isolated from a sample of ropy bread [18] and semifinal gelatin extracts [19]. Recently, Park *et al.* [20] reported the isolation of *B. sonorensis* under anaerobic conditions from a traditionally fermented Korean soya bean paste called eoyukjang. In contrast to Park *et al.* [20], we isolated QQ *B. sonorensis* L62 under aerobic conditions from traditional Chinese soya sauce liquid fermentation.

Palmisano *et al.* [15] described *B. sonorensis* as a novel species that could only be phenotypically distinguished from *B. licheniformis* by salt tolerance and pigmentation. Isolate L62 was salt tolerant and grew optimally in rich media supplemented with 3% (w/v) NaCl (data not shown). Palmisano *et al.* [15] also reported that *B. sonorensis* is an active heterofermenter and a spore-forming bacillus with casein and starch hydrolysis activities. However, isolate L62 did not show positive proteolytic and lipolytic activities (data not shown) but possessed starch hydrolysis activity. It is speculated that *B. sonorensis* L62 may play a role in hydrolysis of the flour introduced during the solid-state (koji) phase fermentation with its amylase.

Palmisano *et al.* [15] suggested using the *rpoB* sequence to distinguish between *B. licheniformis* and *B. sonorensis*. Indeed, the *rpoB* gene sequence of L62 showed closer similarity with that of *B. sonorensis*. Phylogenetic analysis revealed that L62 was closely related to *B. sonorensis*.

Bacilli possessing QQ AHL lactonase (AiiA), similar to that of *Bacillus* sp. 240B1 [7], have been readily isolated from Malaysian rain forest soil by simple heat-treatment of the soil and enrichment with AHL-supplemented KG medium [11,14]. We used published primers [7,11] to amplify the *aiiA* gene. However, no *aiiA* homologue was detected in *B. sonorensis* L62 by PCR (data not shown), suggesting the possibility of a different QQ gene in *B. sonorensis* L62.

*B. sonorensis* L62 showed rapid degradation of AHLs with different C-3 substituents and acyl chain lengths, suggesting broad substrate activity against AHLs. This may represent a nutritional symbiosis in which products of degraded AHLs can be used by other species living in proximity or other well adapted bacteria may act in concert with *B. sonorensis* L62 in the soya sauce fermentation brine to further metabolise AHLs. Moreover, AHLs exist in D- and L-isomers, but our biosensors can only detect the latter. Hence, further work should be done to clone the QQ gene and determine whether the AHLase of *B. sonorensis* L62 is stereospecific against the two isomers of AHLs.

Many AHLs are quite stable in acidic and neutral pH environments [21]. Soya sauce liquid state fermentation is typically characterised by its low pH values (<5) owing largely to the activities of lactobacilli. This low pH environment would not cause AHLs present in the soya sauce fermentation brine to degrade. Therefore, they are most likely degraded by the QQ activities of bacteria such as *B. sonorensis* L62, preventing them from accumulating in soy sauce.

Little is known about the microbial QS and QQ physiotypes during soya sauce liquid state fermentation. Hence, further work should be directed on the role of this QQ *B. sonorensis* L62 in soya sauce fermentation. In addition, we need to isolate QS bacteria from this unique niche and to study them in order to understand the production of AHLs in soya sauce fermentation brine. We also need to determine the concentration of AHLs in soya sauce (if any) as there is no report on the effect of AHLs on humans after consumption. The degradation of AHLs by *B. sonorensis* L62 in the soya sauce mash may provide insights to the role of AHL quenching species, metabolic pathways of AHL turnover in soya sauce mash, and the microbial ecological significance of AHL degradation in soya sauce liquid state fermentation. It is plausible that *B. sonorensis* L62 uses its QQ ability to gain competitive advantage over AHL-dependent signalling bacteria, or *B. sonorensis* L62 is an important bacterium that has vital role in the soya sauce liquid fermentation process.

## 4. Conclusions

Not much is known about the presence of QQ bacteria in the traditional soya sauce fermentation except reports on the presence of halophilic bacteria in this unique fermentation process. This is the first report of a bacterial isolate with broad QQ activity, *B. sonorensis* strain L62, from traditional Chinese soya sauce brine.
